# Single-center retrospective feasibility and implementation analysis of dynamic Caprini assessment-based graded nursing for in-hospital VTE risk management in a meniscoplasty-dominant arthroscopic meniscus surgery cohort

**DOI:** 10.3389/fmed.2026.1838532

**Published:** 2026-08-26

**Authors:** Xiaomei Li, Jimin Ma, Yan Dong

**Affiliations:** 1 Department of Orthopedics, The Fuyang Affiliated Hospital of Anhui Medical University, Fuyang, China; 2 Department of Nursing, The Fuyang Affiliated Hospital of Anhui Medical University, Fuyang, China

**Keywords:** arthroscopic meniscoplasty, Caprini risk assessment, deep vein thrombosis, feasibility, graded nursing, in-hospital venous thromboembolism, symptom-triggered ultrasonography

## Abstract

**Objective:**

To evaluate the feasibility and implementation outcomes of dynamic Caprini assessment-based graded nursing for in-hospital venous thromboembolism (VTE) risk management in a meniscoplasty-dominant arthroscopic meniscus surgery cohort.

**Methods:**

This single-center, retrospective, observational implementation analysis included 90 adult patients who underwent arthroscopic meniscus surgery between January 2024 and December 2025. Caprini assessments were completed at admission (T1), postoperative day 1 (T2), and discharge (T3), and nursing/prophylaxis delivery was assigned according to dynamic risk stratification. Feasibility endpoints included completion of serial assessments, risk-concordant prophylaxis delivery, documentation completeness, and discharge satisfaction. The primary clinical outcome was clinically detected in-hospital deep vein thrombosis (DVT), confirmed by symptom-triggered lower-extremity duplex ultrasonography; pulmonary embolism (PE) was assessed when clinically suspected. Results: Serial Caprini assessment and risk classification were completed in all 90 patients. Basic preventive nursing was delivered to 90/90 patients, mechanical prophylaxis to 73/73 T2 moderate/high-risk patients, and low-molecular-weight heparin (LMWH) to 26/26 T2 high-risk patients. The mean Caprini score increased from T1 (1.87 ± 1.11) to T2 (3.84 ± 1.34, *P* < 0.001) and decreased at T3 (2.68 ± 1.51, *P* < 0.001 vs. T2). Moderate-to-high risk classification increased from 26.7% at T1 to 81.1% at T2. Clinically detected in-hospital DVT occurred in 2/90 patients (2.2%; exact 95% CI, 0.3–7.8%), both distal; PE occurred in 0/90 patients (0%; 95% CI, 0.0–4.0%); and minor bleeding occurred in 1/90 patient (1.1%; 95% CI, 0.0–6.0%). No major bleeding was observed. Mean satisfaction was 98.5 ± 1.2 points.

**Conclusion:**

Dynamic Caprini assessment-based graded nursing was feasible to implement within this single-center meniscoplasty-dominant cohort. The findings describe protocol delivery and clinically detected in-hospital outcomes only and do not establish causal efficacy or total perioperative DVT incidence.

## 1 Introduction

Venous thromboembolism (VTE), encompassing deep vein thrombosis (DVT) and pulmonary embolism (PE), remains a major preventable source of morbidity and mortality among hospitalized surgical patients (1, 2). Although arthroscopic knee surgery is generally regarded as a minimally invasive procedure with a lower thrombotic risk than major orthopedic operations such as joint replacement, accumulating evidence indicates that postoperative VTE after knee arthroscopy is not negligible (3, 4). A meta-analysis reported an overall DVT incidence rate of approximately 9.9% in unprophylaxed patients undergoing knee arthroscopy when routine screening with ultrasound or venography was employed, with proximal DVT detected in 2.1% of cases (4). More recent observational evidence has identified several risk factors for VTE following knee arthroscopy, including advanced age, elevated body mass index (BMI), smoking history, and prolonged tourniquet application (5, 6).

Arthroscopic meniscus surgery, as one of the most commonly performed knee arthroscopic procedures worldwide, presents a specific clinical scenario for risk-stratified VTE management. While the procedure itself is characterized by short operative times and rapid postoperative mobilization, selected patient-related and perioperative factors, such as middle-to-advanced age, obesity, and temporary postoperative mobility limitation, may justify closer thrombotic risk assessment in individual patients (5–7). Current guidelines from the American College of Chest Physicians (ACCP) do not recommend routine pharmacological thromboprophylaxis for all patients undergoing uncomplicated knee arthroscopy, leaving individualized assessment central to clinical decision-making (8). This gap in standardized guidance underscores the need to describe practical risk assessment workflows that can identify patients for whom escalated nursing and prophylactic measures are clinically considered.

The Caprini Risk Assessment Model (RAM), first introduced in 1991 and subsequently validated in broad surgical populations, is one of the most widely adopted tools for individualized VTE risk stratification in surgical patients (2, 9). The model assigns weighted point values to a comprehensive array of patient-specific and procedure-specific risk factors, generating a cumulative score that corresponds to increasing VTE risk. Importantly, the 2013 updated version of the Caprini RAM emphasizes repeated assessment throughout hospitalization, as a patient’s risk profile may evolve in the perioperative period due to surgical stress, immobilization, and other dynamic factors (9, 10). Despite the established utility of the Caprini score in surgical risk stratification, its application in a dynamic, nursing-driven assessment framework specifically tailored to a meniscoplasty-dominant arthroscopic meniscus surgery cohort has not been fully described.

In the context of perioperative nursing, evidence-based and risk-stratified prophylactic protocols represent an important component of patient safety and quality improvement (11, 12). A graded nursing workflow that integrates dynamic Caprini assessment with tiered interventions, ranging from basic prophylaxis through mechanical prophylaxis to pharmacological prophylaxis when clinically indicated, may support standardized documentation and proportionate use of preventive resources (8, 13, 14). However, the feasibility of implementing such an integrated workflow and the associated clinically detected in-hospital outcomes in a meniscoplasty-dominant arthroscopic meniscus surgery population have not been fully described. Therefore, the present study aimed to evaluate feasibility endpoints, protocol delivery, and clinically detected in-hospital DVT/PE outcomes associated with a Caprini dynamic assessment-based graded nursing strategy during the perioperative period at a single orthopedic center.

## 2 Patients and methods

### 2.1 Study design

This was a single-center, retrospective, observational implementation analysis conducted at the Department of Orthopedics, the Fuyang Affiliated Hospital of Anhui Medical University, China. The study period extended from January 1, 2024, to December 31, 2025. The study protocol was approved by the Fuyang Affiliated Hospital of Anhui Medical University ethics committee (approval number: KYPJ-2026-020), and the requirement for individual informed consent was waived due to the retrospective nature of the data collection. All patient data were anonymized prior to analysis, and the study was conducted in accordance with the principles of the Declaration of Helsinki. This investigation followed the Strengthening the Reporting of Observational Studies in Epidemiology (STROBE) guidelines for reporting observational studies (15). The study was designed to describe feasibility, protocol delivery, and clinically detected in-hospital outcomes rather than to establish a causal efficacy effect.

### 2.2 Study participants

Consecutive patients who underwent arthroscopic meniscus surgery at the study institution during the defined study period were screened for eligibility. The inclusion criteria were as follows: (1) confirmed diagnosis of meniscal injury based on clinical examination and magnetic resonance imaging (MRI); (2) surgical treatment with arthroscopic meniscal debridement/meniscoplasty or arthroscopic meniscal repair; and (3) age ≥18 years. The exclusion criteria included: (1) pre-existing DVT confirmed by preoperative duplex ultrasound; (2) known coagulation disorders or ongoing anticoagulant therapy; (3) prior history of VTE within the preceding 12 months; (4) conversion to open surgery during the arthroscopic procedure; and (5) incomplete medical records precluding Caprini score calculation at all three assessment timepoints. During the study period, 104 surgical records were screened and 14 were excluded, including pre-existing DVT (*n* = 2), coagulation disorder or ongoing anticoagulant therapy (*n* = 3), VTE history within 12 months (*n* = 1), conversion to open surgery (*n* = 1), and incomplete Caprini or outcome documentation (*n* = 7). A total of 90 patients were included in the final analysis, comprising 88 patients who underwent arthroscopic meniscoplasty and 2 patients who underwent arthroscopic meniscal repair. No missing values were present in the final dataset for Caprini score, prophylaxis assignment, in-hospital VTE outcome, bleeding event, limb circumference, NRS score, length of stay, or satisfaction score. Operative laterality was not extractable from the anonymized structured operative record in 3 cases; because laterality was not used for Caprini scoring, prophylaxis assignment, or the primary outcome classification, these cases were retained.

### 2.3 Intervention: Caprini dynamic assessment-based graded nursing strategy

#### 2.3.1 Dynamic assessment mechanism

The cornerstone of the nursing intervention was the implementation of serial Caprini Risk Assessment Model (2013 version) evaluations at three predefined perioperative timepoints: (1) T1 (admission assessment), conducted within 2 h of hospital admission by the responsible ward nurse, which established the baseline VTE risk profile by evaluating age-related risk factors, personal and family history of VTE, current medical comorbidities, BMI, mobility status, and any pre-existing risk factors identified from the patient’s medical history and preoperative evaluation; (2) T2 (postoperative assessment), performed within 2 h after the patient returned to the ward by the responsible ward nurse, which incorporated additional surgery-related risk factors including the duration of anesthesia and surgery, the type of arthroscopic procedure performed, the extent of postoperative immobilization, and any new perioperative complications such as surgical site swelling or postoperative infection; and (3) T3 (discharge assessment), completed on the day of planned discharge, which served as the final risk evaluation to determine whether the patient’s risk profile had returned to a level permitting safe discharge without ongoing prophylaxis, or whether extended prophylactic measures were warranted after discharge. All assessments were performed by nursing staff who had completed a standardized 8-h training program on the Caprini RAM, including both theoretical instruction and supervised practical assessment of at least 10 cases before independent scoring. Inter-rater reliability was established through periodic double-scoring of randomly selected cases, with a required agreement threshold of ≥90%.

#### 2.3.2 Risk stratification criteria

Based on the cumulative Caprini score at each assessment timepoint, patients were stratified into three risk categories that guided subsequent prophylactic interventions: (1) low-risk group (Caprini score 0–2 points), indicating minimal VTE risk consistent with the expected baseline for healthy ambulatory individuals undergoing minor procedures; (2) moderate-risk group (Caprini score 3–4 points), indicating an elevated VTE risk that warranted augmented prophylactic measures beyond basic care; and (3) high-risk group (Caprini score ≥5 points), indicating a substantially elevated VTE risk requiring the maximal combination of prophylactic interventions including pharmacological anticoagulation. Critically, because the risk stratification was dynamic and updated at each timepoint, the prophylactic regimen assigned to each patient could be escalated or de-escalated in real-time as the clinical situation evolved throughout the perioperative period. This dynamic approach ensured that patients who transitioned from low risk at admission to moderate or high risk after surgery received timely intensification of prophylaxis, while those whose risk diminished during recovery were not subjected to unnecessary interventions carrying their own inherent risks, such as anticoagulant-associated bleeding.

#### 2.3.3 Graded nursing interventions

The graded nursing intervention protocol was structured into three tiers of escalating intensity, each corresponding to the patient’s current risk classification. The first tier, basic prophylaxis, was applied universally to all patients regardless of risk category and comprised the following measures: (1) elevation of the affected limb to promote venous return and reduce postoperative swelling; (2) active ankle pump exercises and quadriceps contraction training under nursing guidance after surgery; (3) encouragement of early limb movement and early ambulation as tolerated, together with routine position care and activity assistance; and (4) patient and family education regarding the signs and symptoms of DVT and PE, including unilateral limb swelling, calf tenderness, unexplained dyspnea, and pleuritic chest pain, using a standardized Chinese-language educational pamphlet provided at admission and reinforced during hospitalization.

The second tier, enhanced nursing and mechanical prophylaxis, was implemented for patients classified as moderate or high risk (Caprini score ≥3). In addition to the universal basic measures, these patients received closer bedside monitoring and reinforced preventive nursing, including more frequent guidance on lower-limb elevation, ankle pump exercises, quadriceps contraction training, early mobilization, and symptom surveillance for venous thromboembolism. Graduated compression stockings and intermittent pneumatic compression were applied when not contraindicated; mechanical prophylaxis was initiated after the patient returned to the ward and was continued during the in-hospital period, with IPC generally delivered twice daily and stockings used during daytime ambulation or rest according to nursing tolerance. Nursing staff documented the implementation and patient adherence to these preventive measures in the medical record and promptly reported any new swelling, pain, or other warning signs suggestive of thrombotic events.

The third tier, pharmacological prophylaxis, was applied to patients with high thrombotic risk after comprehensive evaluation by the treating team. Low-molecular-weight heparin (LMWH; enoxaparin sodium 4,000 IU or equivalent) was administered according to the patient’s Caprini category, bleeding risk, and perioperative status. In patients without contraindications, LMWH was initiated 6–12 h after surgery once surgical hemostasis was confirmed, continued during hospitalization, and extended after discharge only when persistent high-risk classification or clinical judgment supported continued prophylaxis. Platelet counts and bleeding manifestations were monitored during LMWH administration when clinically indicated to screen for heparin-induced thrombocytopenia and anticoagulant-associated bleeding. Patients not considered suitable for pharmacological anticoagulation received intensified mechanical prophylaxis and close clinical observation (8, 16).

### 2.4 Outcome measures

The primary outcome was clinically detected in-hospital DVT, confirmed by color Doppler ultrasonography of the bilateral lower extremities performed when clinically indicated by symptoms or abnormal clinical findings such as unilateral leg swelling, calf pain, localized tenderness, or elevated D-dimer levels. This endpoint was not defined as total perioperative DVT incidence because universal screening and extended post-discharge ultrasound surveillance were not performed. PE was recorded when confirmed by computed tomography pulmonary angiography (CTPA) in patients with clinical suspicion such as unexplained dyspnea, chest pain, hypoxemia, or hemodynamic instability. Feasibility endpoints were operationalized as completion of Caprini assessment at all three timepoints, successful risk-category assignment at each timepoint, delivery and documentation of basic prophylaxis to all included patients, risk-concordant mechanical prophylaxis among patients classified as moderate or high risk at T2, risk-concordant LMWH delivery among patients classified as high risk at T2, documentation completeness for in-hospital VTE and bleeding outcomes, and discharge nursing satisfaction. Secondary outcome measures included dynamic changes in Caprini scores and risk stratification across the three assessment timepoints; limb circumference changes, measured by a trained nurse using a nonelastic measuring tape at 10 cm above the superior border of the patella and at 10 cm below the tibial tubercle, with results expressed as the difference between the operative and contralateral limbs at the preoperative, postoperative day 1, and discharge timepoints; pain intensity assessed by trained ward nurses using the NRS at the same three timepoints; length of hospital stay; bleeding complications, defined as any clinically significant bleeding event requiring treatment modification, local intervention, transfusion, or additional observation; and patient satisfaction with nursing care, assessed at discharge using a standardized 100-point questionnaire covering explanation of DVT prevention, timeliness of nursing response, exercise guidance, pain and swelling care, and discharge education. Outcome measurements were performed according to the department’s standardized nursing quality-control procedure; limb circumference and NRS assessments were completed by trained ward nurses, and discharge questionnaires were administered and checked by the responsible discharge nurse before the patient left the ward.

### 2.5 Statistical analysis

Statistical analyses were performed using SPSS version 26.0 (IBM Corp., Armonk, NY, United States). Continuous variables were tested for normality using the Shapiro-Wilk test and expressed as mean ± standard deviation (SD) for normally distributed data or as median (interquartile range) for non-normally distributed data. Categorical variables were presented as frequencies and percentages. Exact two-sided 95% confidence intervals (CIs) for rare binary outcomes and feasibility proportions were calculated using the Clopper-Pearson method. Caprini scores, limb circumference differences, and NRS scores across three paired timepoints were analyzed using repeated-measures analysis of variance (ANOVA) with Bonferroni post-hoc correction when distributional assumptions were satisfied, or the Friedman test with Wilcoxon signed-rank post-hoc tests when assumptions were not satisfied. Pairwise comparisons between consecutive timepoints were conducted using paired *t*-tests or Wilcoxon signed-rank tests as appropriate. Risk category transitions across the three paired ordinal categories were evaluated using marginal homogeneity methods, including the Stuart-Maxwell test for global paired category changes and Bowker symmetry testing for transition matrices; dichotomized low-risk versus moderate-to-high-risk comparisons were evaluated with exact McNemar tests when needed. Exploratory correlation analyses were limited to supplementary descriptive visualization and used Pearson correlation coefficients. All statistical tests were two-tailed, and a *P* < 0.05 was considered statistically significant (17).

## 3 Results

### 3.1 Baseline characteristics

**FIGURE 1:**
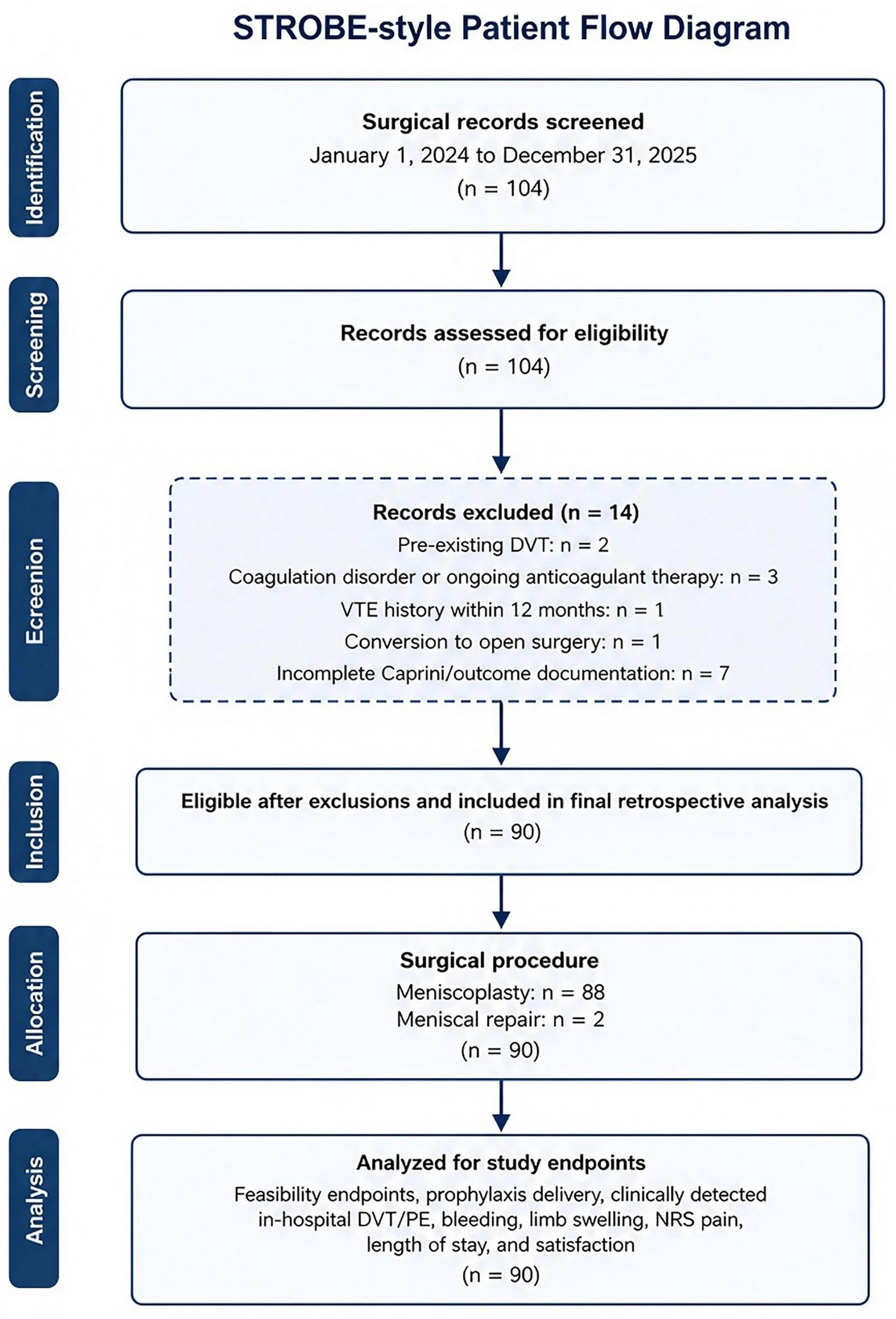
STROBE-style flow diagram showing patient screening, exclusion, and final inclusion in the retrospective analysis. During the study interval, 104 surgical records were screened; 14 records were excluded because of pre-existing DVT, coagulation disorder or ongoing anticoagulant therapy, recent VTE history, conversion to open surgery, or incomplete Caprini/outcome documentation; and 90 patients were included in the final analysis.

During the study interval, 104 records were screened, 14 were excluded, and 90 patients were included in the final analysis after application of the exclusion criteria; the STROBE-style flow diagram is shown in Figure 1. The baseline demographic and clinical characteristics are presented in Table 1. The study cohort comprised 44 males (48.9%) and 46 females (51.1%), with a mean age of 50.3 ± 11.9 years. The mean BMI was 25.2 ± 3.1 kg/m^2^. The cohort was meniscoplasty-dominant: 88 patients (97.8%) underwent arthroscopic meniscoplasty, whereas 2 patients (2.2%) underwent arthroscopic meniscal repair. The operative side was right in 50 cases (55.6%), left in 35 cases (38.9%), bilateral in 2 cases (2.2%), and unspecified in 3 cases (3.3%) because laterality was unavailable in the anonymized structured operative record. The mean surgical duration was 64.0 ± 13.8 min, the mean preoperative D-dimer level was 0.44 ± 0.23 mg/L, and the mean length of hospital stay was 5.5 ± 1.8 days.

**TABLE 1:** Baseline demographic and clinical characteristics (*n* = 90).

Variable	Value
Age (years), mean ± SD	50.3 ± 11.9
Sex, n (%)	
Male	44 (48.9)
Female	46 (51.1)
BMI (kg/m^2^), mean ± SD	25.2 ± 3.1
Surgical procedure, n (%)	
Arthroscopic meniscoplasty	88 (97.8)
Arthroscopic meniscal repair	2 (2.2)
Operative side, n (%)	
Right	50 (55.6)
Left	35 (38.9)
Bilateral	2 (2.2)
Unspecified	3 (3.3)
Surgical duration (min), mean ± SD	64.0 ± 13.8
Preoperative D-dimer (mg/L), mean ± SD	0.44 ± 0.23
Length of stay (days), mean ± SD	5.5 ± 1.8

SD, standard deviation; BMI, body mass index.

### 3.2 Dynamic changes in Caprini scores and risk stratification

**FIGURE 2:**
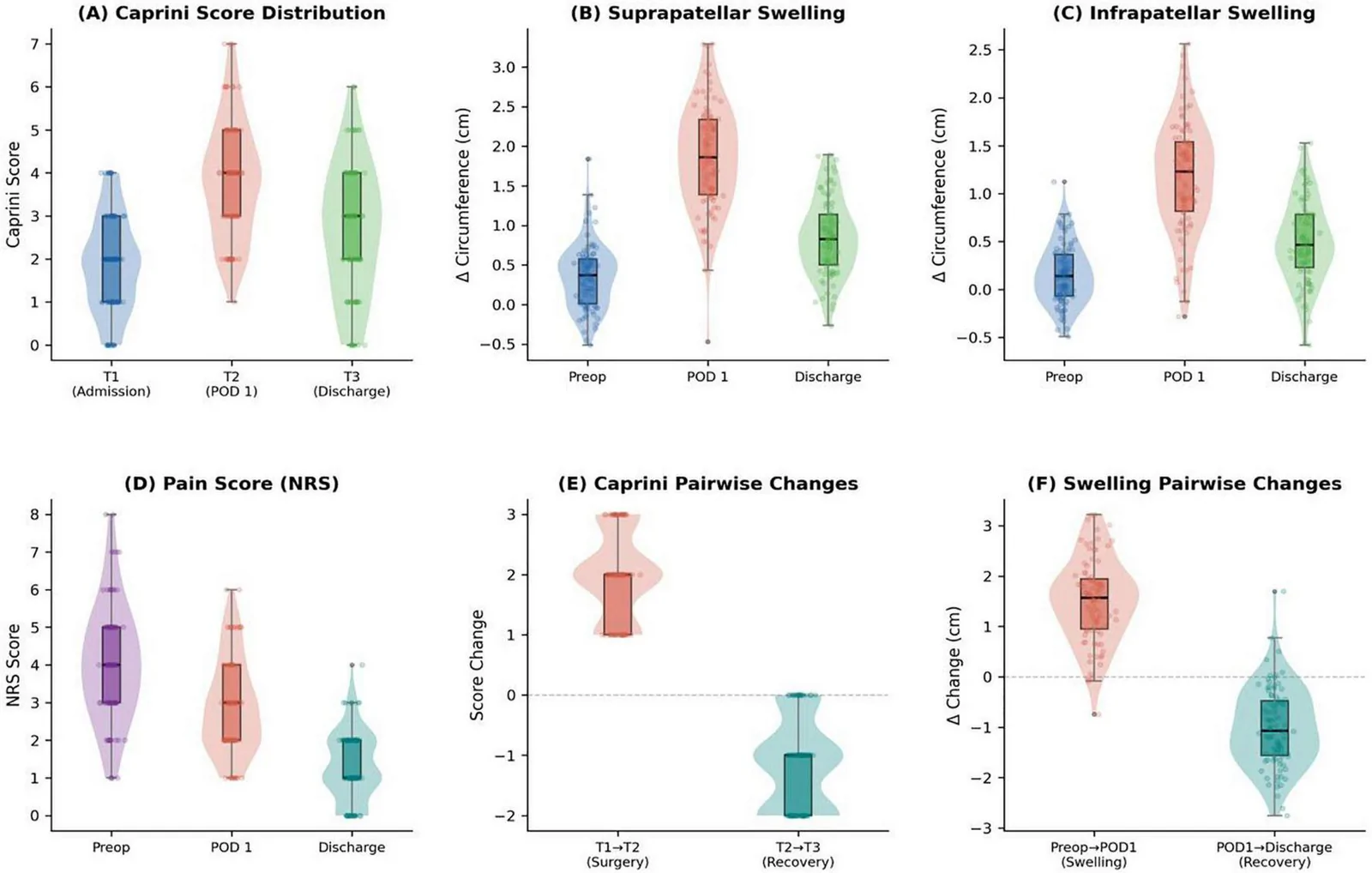
Violin-box-swarm plots showing perioperative outcome distributions. **(A)** Caprini score at T1, T2, and T3; **(B)** suprapatellar circumference difference; **(C)** infrapatellar circumference difference; **(D)** NRS pain scores; **(E)** Caprini pairwise score changes (T1→T2 vs. T2→T3); **(F)** limb swelling pairwise changes (Preop→POD1 vs. POD1→Discharge). Violin shading represents distribution density; box plots indicate median and interquartile range; individual data points are overlaid.

The Caprini scores demonstrated significant dynamic changes across the three perioperative assessment timepoints. The mean Caprini score at admission (T1) was 1.87 ± 1.11, increased to 3.84 ± 1.34 at postoperative day 1 (T2; paired *t*-test, *t* = −26.111, *P* < 0.001), and decreased to 2.68 ± 1.51 at discharge (T3; paired comparison vs. T2, *t* = 14.693, *P* < 0.001), while remaining significantly higher than baseline (*P* < 0.001). These changes reflected the addition and subsequent partial resolution of surgery-related risk factors. The distributions of Caprini scores, limb swelling, and NRS pain at each timepoint are displayed in Figure 2.

**FIGURE 3:**
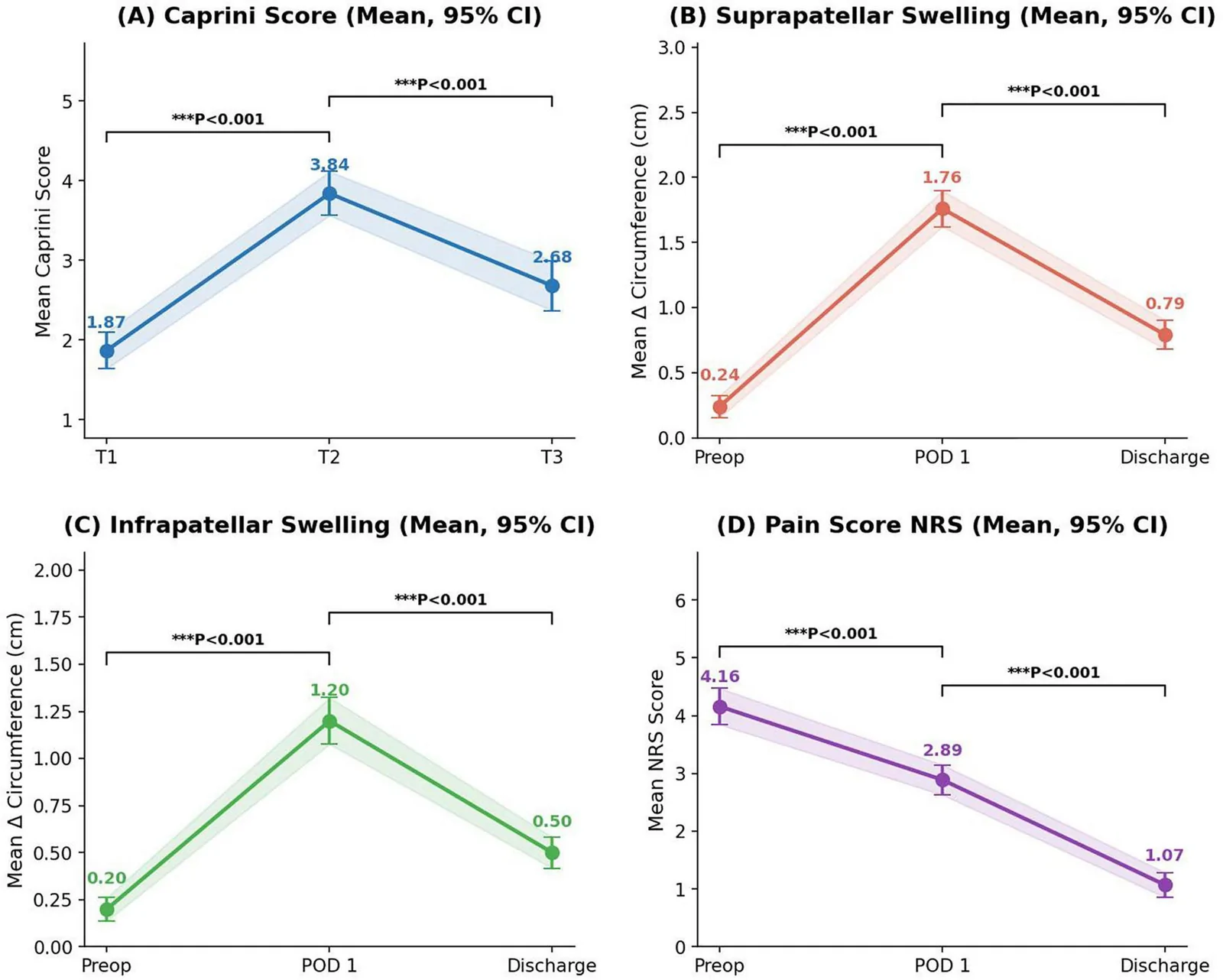
Mean trend lines with 95% confidence intervals across perioperative timepoints. **(A)** Caprini score; **(B)** suprapatellar circumference difference; **(C)** infrapatellar circumference difference; **(D)** NRS pain score. The plotted means correspond to the values reported in Tables 2, 4. Error bars represent 95% CI; significance brackets indicate ***P < 0.001 between consecutive timepoints.

The group-level mean trends with 95% confidence intervals and statistical significance annotations are presented in Figure 3. The plotted means correspond to the summary statistics for the Caprini score, suprapatellar circumference difference, infrapatellar circumference difference, and NRS pain score. All four measures demonstrated statistically significant changes between consecutive timepoints (all *P* < 0.001), indicating a dynamic perioperative clinical course while not implying a causal intervention effect.

**FIGURE 4:**
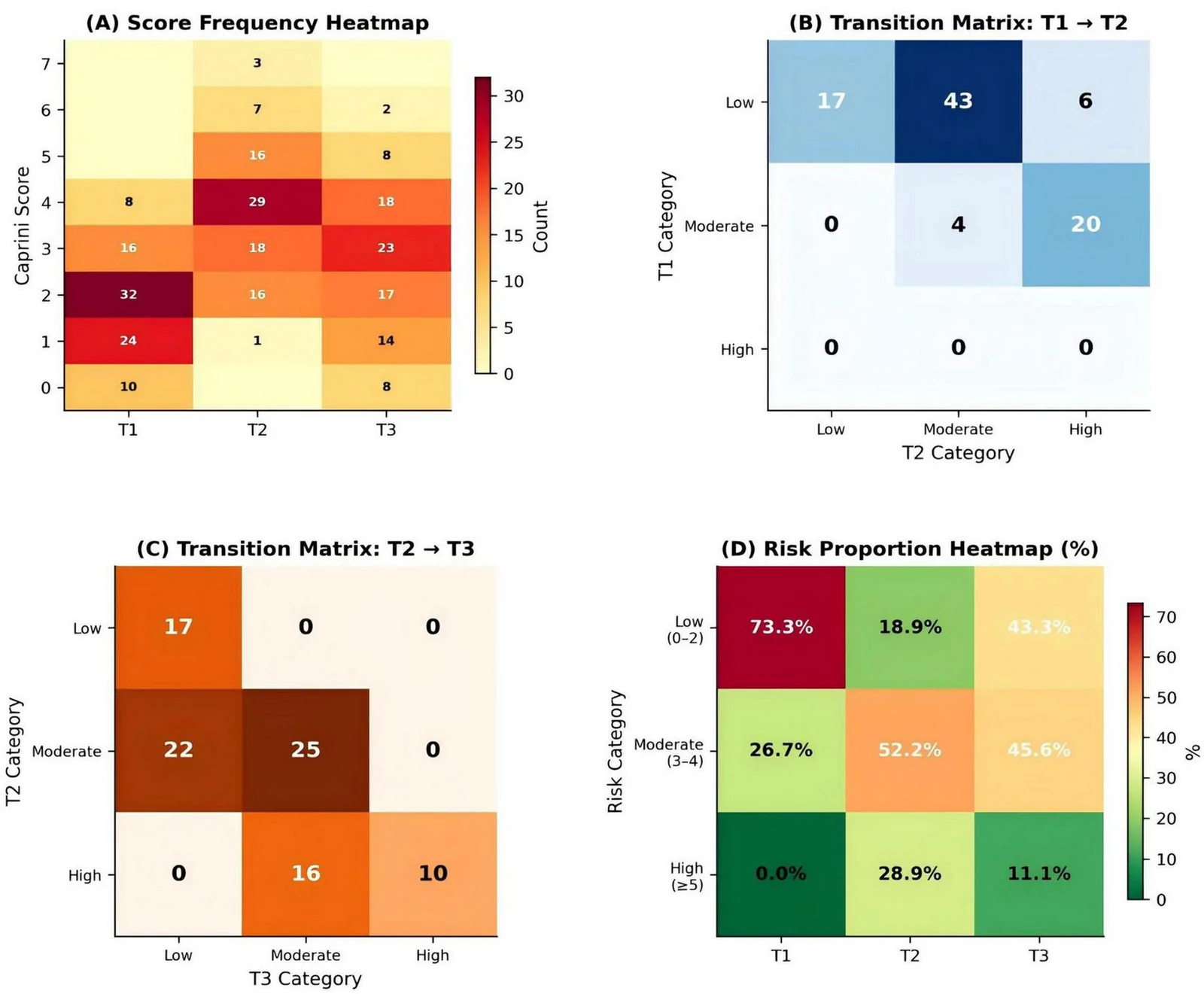
Heatmap analysis of Caprini score distributions and risk stratification transitions. **(A)** Score frequency heatmap showing the number of patients at each Caprini score across T1–T3; **(B)** transition matrix from T1 to T2 displaying patient flow between risk categories; **(C)** transition matrix from T2 to T3; **(D)** risk proportion heatmap showing the percentage of patients in each risk category across timepoints.

The risk stratification patterns shifted markedly across the perioperative period (Figure 4 and Table 2). At T1, the majority of patients were classified as low risk (66/90, 73.3%), with 24 patients (26.7%) in the moderate-risk category and no patients in the high-risk category. Following surgery (T2), only 17 patients (18.9%) remained in the low-risk category, while 47 (52.2%) were classified as moderate risk and 26 (28.9%) as high risk. The transition matrices (Figures 4B,C) showed that 43 of 66 T1-low-risk patients transitioned to moderate risk and 6 to high risk at T2, whereas by T3, 22 of 47 T2-moderate-risk patients returned to low risk. By discharge (T3), 39 patients (43.3%) were low risk, 41 (45.6%) were moderate risk, and 10 (11.1%) remained high risk. Marginal homogeneity testing indicated significant paired category shifts from T1 to T2 and from T2 to T3 (both *P* < 0.001), supporting serial reassessment and flexible prophylactic nursing assignment.

**TABLE 2:** Caprini scores and risk stratification at three perioperative timepoints.

Variable	T1	T2	T3
Caprini score, mean ± SD	1.87 ± 1.11	3.84 ± 1.34*	2.68 ± 1.51*^†^
Low risk (0–2), *n* (%)	66 (73.3)	17 (18.9)	39 (43.3)
Moderate risk (3–4), *n* (%)	24 (26.7)	47 (52.2)	41 (45.6)
High risk ( ≥5), *n* (%)	0 (0)	26 (28.9)	10 (11.1)

*P < 0.001 vs. T1;

† *P* < 0.001 vs. T2 for the Caprini score. Risk-category transitions were evaluated as paired ordinal categorical data using Stuart-Maxwell marginal homogeneity and Bowker symmetry tests. SD, standard deviation.

### 3.3 VTE Prevention outcomes

All 90 patients completed Caprini assessment and risk-category assignment at T1, T2, and T3, and no missing values were present for the feasibility endpoints, prophylaxis delivery variables, or in-hospital VTE/bleeding outcomes. Basic preventive nursing measures were documented for all 90 patients (100.0%; exact 95% CI, 96.0–100.0%). Mechanical prophylaxis with graduated compression stockings and/or intermittent pneumatic compression was delivered to all 73 patients classified as moderate or high risk at T2 (73/73, 100.0%; exact 95% CI, 95.1–100.0%). LMWH prophylaxis was delivered to all 26 patients classified as high risk at T2 (26/26, 100.0%; exact 95% CI, 86.8–100.0%), and these 26 patients were a subset of the 73 patients receiving mechanical prophylaxis. LMWH was initiated 6–12 h after surgery after confirmation of hemostasis and continued during hospitalization; patients who remained high risk at discharge were evaluated for short-term extended prophylaxis. Clinically detected in-hospital DVT was diagnosed in 2/90 patients (2.2%; exact 95% CI, 0.3–7.8%), both of whom presented with distal calf vein thrombosis detected by color Doppler ultrasonography after onset of mild unilateral calf swelling and tenderness. Both affected patients were in the high-risk category at T2, with Caprini scores of 6 and 7, respectively. The DVT episodes were managed with therapeutic anticoagulation (LMWH followed by oral rivaroxaban for 3 months), and neither case showed documented progression to proximal DVT or PE during hospitalization. PE was observed in 0/90 patients (0%; exact 95% CI, 0.0–4.0%). One patient (1/90, 1.1%; exact 95% CI, 0.0–6.0%) experienced minor subcutaneous ecchymosis at the LMWH injection site that did not require discontinuation of anticoagulation or additional intervention. No major bleeding was observed. The feasibility endpoints, prophylaxis delivery, and clinically detected in-hospital VTE/bleeding outcomes are summarized in Table 3.

**TABLE 3:** Feasibility endpoints, prophylaxis delivered, and clinically detected in-hospital VTE/complication outcomes (*n* = 90).

Item	n/N (%) or protocol detail
Caprini assessment completed at T1, T2, and T3	90/90 (100.0%; 95% CI, 96.0–100.0)
Risk-category assignment completed at all timepoints	90/90 (100.0%; 95% CI, 96.0–100.0)
Basic prophylaxis delivered	90/90 (100.0%; 95% CI, 96.0–100.0)
Mechanical prophylaxis delivered among T2 moderate/high-risk patients	73/73 (100.0%; 95% CI, 95.1–100.0)
LMWH prophylaxis delivered among T2 high-risk patients	26/26 (100.0%; 95% CI, 86.8–100.0)
LMWH initiation and duration	6–12 h postoperatively; during hospitalization
Persistent high risk at discharge assessed for extension	10/90 (11.1%; 95% CI, 5.5–19.5)
Clinically detected in-hospital DVT (total)	2/90 (2.2%; 95% CI, 0.3–7.8)
Distal DVT	2/90 (2.2%; 95% CI, 0.3–7.8)
Proximal DVT	0/90 (0%; 95% CI, 0.0–4.0)
Pulmonary embolism during hospitalization	0/90 (0%; 95% CI, 0.0–4.0)
Clinically detected in-hospital VTE	2/90 (2.2%; 95% CI, 0.3–7.8)
Any bleeding complication	1/90 (1.1%; 95% CI, 0.0–6.0)
Major bleeding	0/90 (0%; 95% CI, 0.0–4.0)
Minor bleeding (injection site ecchymosis)	1/90 (1.1%; 95% CI, 0.0–6.0)

CI, confidence interval; DVT, deep vein thrombosis; GCS, graduated compression stockings; IPC, intermittent pneumatic compression; LMWH, low-molecular-weight heparin; VTE, venous thromboembolism. Exact 95% CIs were calculated using the Clopper-Pearson method for binary proportions. DVT denotes clinically detected in-hospital DVT, not total perioperative DVT incidence.

### 3.4 Limb swelling and pain improvement

The limb circumference difference between the operative and contralateral limbs demonstrated a characteristic postoperative pattern of initial swelling followed by partial resolution by discharge (Figures 2B,C, 3B,C). At the suprapatellar measurement site, the mean circumference difference increased from 0.24 ± 0.41 cm preoperatively to 1.76 ± 0.67 cm on postoperative day 1 (*P* < 0.001), and subsequently decreased to 0.79 ± 0.54 cm at discharge (*P* < 0.001 vs. POD 1). At the infrapatellar measurement site, the corresponding values were 0.20 ± 0.30 cm, 1.20 ± 0.60 cm, and 0.50 ± 0.40 cm at the preoperative, POD 1, and discharge timepoints, respectively (all pairwise *P* < 0.001). Exploratory scatter plots were moved to Supplementary Figure 1 and were not used for primary inference.

Pain assessment using the NRS showed a progressive and statistically significant reduction across the perioperative period (Figures 2D, 3D). The mean NRS score decreased from 4.16 ± 1.52 preoperatively to 2.89 ± 1.26 on postoperative day 1 (*P* < 0.001) and further to 1.07 ± 1.05 at discharge (*P* < 0.001 vs. both preoperative and POD 1 values). Individual NRS trajectories showed consistent progressive improvement. Subgroup analysis by dynamic risk classification showed comparable discharge NRS scores across risk groups, while length of stay also did not differ significantly between risk categories. These results are presented in Table 4.

**TABLE 4:** Perioperative changes in limb swelling and pain scores.

Variable	Preoperative	POD 1	Discharge
Suprapatellar Δ (cm)	0.24 ± 0.41	1.76 ± 0.67*	0.79 ± 0.54*^†^
Infrapatellar Δ (cm)	0.20 ± 0.30	1.20 ± 0.60*	0.50 ± 0.40*^†^
NRS pain score	4.16 ± 1.52	2.89 ± 1.26*	1.07 ± 1.05*^†^

*P < 0.001 vs. Preoperative;

† *P* < 0.001 vs. POD 1. Values are consistent with Figure 3. Δ, circumference difference (operative - contralateral limb); NRS, Numeric Rating Scale; POD, postoperative day.

### 3.5 Feasibility, patient satisfaction, and subgroup analysis

Feasibility endpoints were met for assessment completion, risk-category assignment, risk-concordant prophylaxis delivery, and complete documentation of in-hospital VTE/bleeding outcomes. Patient satisfaction with nursing care was high, with a mean score of 98.5 ± 1.2 points out of 100 at discharge. The satisfaction instrument consisted of five standardized 20-point domains assessing explanation of DVT prevention, responsiveness of nursing care, guidance on ankle pump and quadriceps exercises, pain and swelling care, and discharge education; higher scores indicated greater satisfaction. The questionnaire was administered before discharge as part of routine nursing quality assessment, and all 90 patients (100.0%; exact 95% CI, 96.0–100.0%) achieved a satisfaction score of ≥95 points. In the subgroup analysis comparing patients who remained low risk at all three timepoints (*n* = 17) with those reclassified to moderate or high risk at any timepoint (*n* = 73), no statistically significant differences were observed in length of hospital stay (5.2 ± 1.5 vs. 5.6 ± 1.9 days, *P* = 0.428) or discharge NRS scores (0.94 ± 0.90 vs. 1.10 ± 1.08, *P* = 0.562). Both clinically detected in-hospital DVT events occurred in the moderate-to-high risk subgroup (2/73, 2.7%), while no DVT events were observed in the persistently low-risk subgroup (0/17, 0%); this difference did not reach statistical significance because of the small number of events (Fisher’s exact test, *P* = 1.000). These exploratory subgroup results are summarized in Table 5.

**TABLE 5:** Exploratory subgroup analysis by dynamic risk reclassification.

Group	*n*	Length of stay (days)	Discharge NRS score	Clinically detected DVT
Persistently low risk	17	5.2 ± 1.5	0.94 ± 0.90	0 (0)
Reclassified to moderate/high risk	73	5.6 ± 1.9	1.10 ± 1.08	2 (2.7)
*P*-value	–	0.428	0.562	1.000

DVT, deep vein thrombosis; NRS, Numeric Rating Scale. *P-*values were calculated using the independent-samples *t*-test or Mann-Whitney U test for continuous variables as appropriate and Fisher’s exact test for DVT events. Values are n, mean ± SD, or n (%).

## 4 Discussion

The present study should be interpreted as a retrospective feasibility and implementation analysis of a Caprini dynamic assessment-based graded nursing strategy in a meniscoplasty-dominant cohort of patients undergoing arthroscopic meniscus surgery. The clinically detected in-hospital DVT rate was 2.2% (exact 95% CI, 0.3–7.8%), with no observed PE (0%; exact 95% CI, 0.0–4.0%) and no major bleeding during hospitalization. Because DVT assessment was symptom-triggered rather than based on universal screening and because follow-up was limited to the in-hospital period, this rate should not be directly compared with screening-based estimates that include asymptomatic DVT or studies with extended post-discharge surveillance (3, 4, 18). The findings therefore support the practical feasibility and short-term safety monitoring of this nursing workflow, but they do not establish that the strategy caused a reduction in DVT incidence compared with standard care. This distinction is particularly important because current guideline recommendations for routine knee arthroscopy remain conservative and emphasize individualized risk assessment rather than routine pharmacological prophylaxis for all patients (8).

A central finding of this study is the substantial dynamic shift in VTE risk stratification observed across the perioperative period, which underscores the rationale for serial rather than single-timepoint risk assessment. At admission, the majority of patients (73.3%) were classified as low risk, reflecting the generally favorable baseline characteristics of patients undergoing elective arthroscopic meniscus surgery. After surgery, the proportion of moderate-to-high risk patients increased to 81.1%, driven by the accumulation of surgery-related risk factors including anesthesia exposure, operative immobilization, and soft-tissue trauma. This finding is consistent with the established understanding that the Caprini score should be recalculated postoperatively to capture the evolving perioperative risk profile (11, 12), and it highlights the potential inadequacy of a single preoperative assessment for guiding prophylactic decisions in this setting.

The graded nursing framework translated dynamic Caprini results into a structured protocol that could be implemented within routine ward practice. In the final cohort, all 90 patients received basic preventive nursing, 73 T2 moderate/high-risk patients received mechanical prophylaxis, and 26 T2 high-risk patients received LMWH after evaluation of bleeding risk. This tiered delivery pattern is conceptually aligned with recommendations for risk-proportionate prophylaxis, in which the intensity of preventive measures is matched to the patient’s thrombotic and hemorrhagic risk (8, 16). A systematic review by Kakkos and colleagues demonstrated that combined intermittent pneumatic compression and pharmacological prophylaxis reduced PE compared with pharmacological prophylaxis alone in high-risk surgical patients (19). Similarly, evidence from other surgical populations suggests that layered mechanical prophylaxis may add benefit compared with a single mechanical modality (20). In the present study, these external findings provide theoretical support for the graded approach, but the single-arm design prevents attribution of the observed in-hospital event rate to the intervention itself.

The observed pattern of limb swelling, peaking on postoperative day 1 and partially resolving by discharge, is consistent with the expected inflammatory response to arthroscopic surgery and with the perioperative course documented in Table 4 and Figure 3. The reduction in NRS pain scores from the preoperative period to discharge reflects the observed postoperative recovery trajectory under standardized nursing care, early mobilization, and analgesic management. The high discharge satisfaction scores may reflect the structured patient education and repeated nursing contact embedded in the protocol, particularly the explanation of DVT warning signs, exercise instruction, and discharge guidance. These secondary outcomes should be interpreted as descriptive implementation and recovery measures rather than independent evidence of thrombosis-prevention efficacy.

Risk factors for VTE following knee arthroscopy have been increasingly studied. Recent observational syntheses have identified age, BMI, smoking history, oral contraceptive use, and prolonged tourniquet use as relevant risk factors for VTE following arthroscopic knee surgery (5–7). In the present cohort, both patients with clinically detected in-hospital DVT were classified as high risk at T2, with Caprini scores of 6 and 7, supporting the clinical plausibility of the dynamic risk stratification framework. Exploratory linear regression plots were retained as Supplementary Figure 1 only; their weak and statistically non-significant correlations were considered descriptive and were not used to support primary conclusions.

Several methodological considerations merit discussion when interpreting the results. First, the single-center, retrospective, observational design and absence of a concurrent standard-care control group prevent causal inference regarding prophylactic efficacy. Second, the sample size of 90 patients limits statistical power for rare outcomes such as DVT and PE, as reflected by the wide exact 95% CIs, and subgroup analyses should be considered exploratory. Third, the primary endpoint was clinically detected in-hospital DVT; symptom-triggered ultrasound may underdetect asymptomatic events, and the lack of standardized post-discharge surveillance means that delayed DVT events could not be fully captured. Previous studies using routine ultrasonographic screening have reported substantially higher DVT rates in knee arthroscopy populations (3, 4), underscoring the need to distinguish symptomatic from asymptomatic DVT, screening-based from symptom-triggered detection, and in-hospital from extended follow-up incidence. Fourth, generalizability is limited because 88 of 90 patients underwent arthroscopic meniscoplasty, with only 2 undergoing meniscal repair; therefore, the findings primarily apply to a meniscoplasty-dominant arthroscopic meniscus surgery cohort rather than all arthroscopic meniscus procedures.

Despite these limitations, the study has several strengths, including use of a validated and internationally recognized risk assessment tool, implementation of a standardized nursing protocol with explicit documentation requirements, operationalized feasibility endpoints, and inclusion of multiple clinically relevant implementation and recovery outcomes. Future studies should employ prospective, multicenter controlled designs comparing the Caprini-based graded nursing strategy with standard care or alternative risk assessment tools, with universal DVT screening by duplex ultrasonography at standardized timepoints and post-discharge follow-up to capture both symptomatic and asymptomatic events. Such studies would improve generalizability, increase power for rare outcomes such as PE, and allow evaluation of health-economic questions related to serial Caprini assessment and tiered prophylactic resource use (21).

## 5 Conclusion

In conclusion, this retrospective single-arm study supports the feasibility of implementing a Caprini dynamic assessment-based graded nursing strategy for in-hospital VTE risk management in a meniscoplasty-dominant cohort of patients undergoing arthroscopic meniscus surgery. Serial Caprini assessment captured substantial perioperative changes in risk category and provided a structured basis for escalation and de-escalation of nursing and prophylactic measures. The observed clinically detected in-hospital DVT rate of 2.2% (exact 95% CI, 0.3–7.8%), absence of PE and major bleeding, perioperative changes in swelling and pain scores, and high discharge satisfaction describe short-term implementation outcomes. Because the study lacked a concurrent control group, used symptom-triggered DVT detection, and had no extended post-discharge screening, the findings should be considered preliminary and should not be interpreted as causal efficacy or broad generalizability beyond similar meniscoplasty-dominant arthroscopic cohorts.

## Data Availability

The original contributions presented in this study are included in this article/Supplementary material, further inquiries can be directed to the corresponding authors.

## Supplementary material

Supplementary Figure 1 Scatter plots with exploratory linear regression analysis of key correlations. **(A)** Patient age vs. T2 Caprini score; **(B)** BMI vs. T2 Caprini score; **(C)** suprapatellar vs. infrapatellar circumference difference at POD 1; **(D)** age vs. discharge NRS score. The annotated *r*- and *P*-values indicate weak, statistically non-significant correlations in all panels; regression lines are fitted by ordinary least squares for descriptive visualization only.
